# Mineral Bone Disorders in Chronic Kidney Disease Stages 3 and 4 With Special Reference to Bone Mineral Density Assessment Using Dual-Energy X-Ray Absorptiometry (DEXA) Scan: A Prospective Observational Study From Northeast India

**DOI:** 10.7759/cureus.110037

**Published:** 2026-06-01

**Authors:** Prodip K Doley, Angelia L Khawbung, Manjuri Sharma, Gayatri Pegu, Miranda Pegu

**Affiliations:** 1 Nephrology, Gauhati Medical College and Hospital, Guwahati, IND

**Keywords:** bone mineral density, chronic kidney disease, ckd-mbd, dexa, osteoporosis, vitamin d deficiency

## Abstract

Background

Chronic kidney disease-mineral and bone disorder (CKD-MBD) is a systemic complication characterized by biochemical disturbances in calcium, phosphate, parathyroid hormone (PTH), and vitamin D metabolism. These abnormalities impair bone strength and promote vascular calcification, increasing fracture and cardiovascular risk. Early CKD stages (3 and 4) provide an opportunity for detection and intervention. Data on Indian pre-dialysis populations remain limited.

Objectives

This study aimed to describe the clinical and biochemical characteristics of CKD-MBD in patients with CKD stages 3 and 4 and to assess bone mineral density (BMD) using dual-energy X-ray absorptiometry (DEXA) scan.

Methodology

This single-centre observational cross-sectional study was conducted in the Department of Nephrology, Gauhati Medical College, Guwahati, Assam, India, from May 2024 to April 2025. A total of 130 pre-dialysis CKD patients (Stage 3: n=72; Stage 4: n=58) were enrolled. Baseline demographic variables, comorbidities, clinical presentation, biochemical parameters (serum calcium, phosphate, intact parathyroid hormone (iPTH), 25-hydroxyvitamin D), and lumbar spine and bilateral femoral sites (right and left femur) were assessed using DEXA scan. BMD categories were classified as normal, osteopenia, or osteoporosis according to WHO criteria. A p-value <0.05 was considered statistically significant. Statistical analysis was performed using SPSS software.

Results

The mean age of the participants was 47.9±14.3 years; 59.2% were men. Compared to Stage 3, Stage 4 patients had significantly higher creatinine (2.86±0.46 vs 1.90±0.29 mg/dL, p<0.001), lower vitamin D (21.4±6.8 vs 32.2±7.6 ng/mL, p<0.0001), higher iPTH (329.7±92.2 vs 274.2±84.1 pg/mL, p=0.0006), and higher phosphate levels (p=0.0011). DEXA revealed lower T-scores in Stage 4 at all sites (p≤0.0004). Osteoporosis prevalence was 44.8% in Stage 4 versus 18.1% in Stage 3 (p=0.0014).

Conclusion

CKD-MBD begins early in stages 3 and 4, with progressive biochemical derangements and significant bone loss. The study demonstrated an association between advancing CKD stage and lower bone mineral density. While DEXA may aid in bone health assessment in CKD patients, further longitudinal studies are needed to establish its predictive and screening utility.

## Introduction

Chronic kidney disease (CKD) is a long-term condition in which kidney function declines gradually and irreversibly [1-3]. Its global burden continues to rise, largely because of diabetes, hypertension, and population ageing [1-3].

One of the major systemic complications of CKD is chronic kidney disease-mineral and bone disorder (CKD-MBD) [4,5]. It is characterized by disturbances in calcium, phosphate, parathyroid hormone, and vitamin D homeostasis, leading to abnormalities in bone turnover, mineralization, volume, and strength, along with vascular and soft-tissue calcification [4,5].

Kidney Disease: Improving Global Outcomes (KDIGO) defines CKD-MBD as a systemic disorder of mineral and bone metabolism due to CKD, manifested by abnormalities in biochemical parameters, bone histology, or vascular and soft-tissue calcification [6]. In CKD, phosphate retention, reduced vitamin D activation, hypocalcaemia, and secondary hyperparathyroidism can begin before dialysis and contribute to skeletal disease [7,8]. These abnormalities are associated with increased risks of fragility fractures, cardiovascular calcification, hospitalization, reduced quality of life, and mortality in CKD patients. Early identification of CKD-MBD is therefore essential to improve long-term skeletal and cardiovascular outcomes.

In CKD stages 3 and 4, clinically obvious skeletal deformity is uncommon, but early reductions in bone strength and increasing fracture risk may already be present [9]. Recognizing skeletal involvement at this stage creates an opportunity for earlier evaluation and intervention before progression to advanced kidney failure. Dual-energy X-ray absorptiometry (DEXA) is the most widely used imaging method for measuring bone mineral density in clinical practice [10,11]. However, in CKD-MBD, DEXA has important limitations because it cannot reliably differentiate abnormalities of bone turnover or characterize the underlying type of renal osteodystrophy. Therefore, interpretation of DEXA findings in isolation may be insufficient, and correlation with biochemical markers of CKD-MBD may provide a more clinically meaningful assessment of skeletal involvement in predialysis CKD.

Data on CKD-MBD in pre-dialysis Indian populations are still limited. Indian studies have shown a high burden of biochemical abnormalities in CKD, but combined evaluation of biochemical markers and DEXA-based skeletal status in CKD stages 3 and 4 remains insufficiently described [12,13]. Moreover, evidence from Northeast India is particularly scarce despite potential regional differences in nutritional status, dietary patterns, sunlight exposure, socioeconomic factors, and healthcare access that may influence mineral metabolism and bone health in CKD patients.

This study aimed to explore the range of mineral and bone disorders present in patients with CKD stages 3 and 4 within a cohort from Northeast India. Particular focus was placed on the evaluation of bone mineral density using dual-energy X-ray absorptiometry (DEXA) scans. By characterizing the prevalence and patterns of mineral abnormalities and reduced BMD in this population, this study seeks to enhance the understanding of CKD-MBD in this region and underscore the importance of early screening and management to mitigate skeletal complications.

This article was previously presented as a poster at the 2026 World Congress of Nephrology (WCN), Japan, on March 29, 2026.

## Materials and methods

Study design and settings

This single-centre cross-sectional observational study with prospective recruitment was conducted in the Department of Nephrology, Gauhati Medical College, Guwahati, Assam, India, from May 2024 to April 2025. It evaluated the clinical and biochemical profile of CKD-MBD in patients with CKD stages 3 and 4 and assessed bone mineral density using DEXA.

Study population and sampling

Adult patients aged 18 years or older with CKD stages 3 and 4 were enrolled from the outpatient and inpatient services. CKD stage 3 and stage 4 were defined by an estimated glomerular filtration rate (eGFR) values of 30-59 and 15-29 mL/min/1.73 m², respectively, calculated using the 2021 Chronic Kidney Disease Epidemiology Collaboration (CKD-EPI) creatinine equation [14]. Patients who had not received calcium supplements, vitamin D analogues, phosphate binders, or calcimimetics for at least three months before enrolment were also included.

Patients on maintenance hemodialysis or continuous ambulatory peritoneal dialysis (CAPD) and those who did not provide consent were excluded.

Data collection and measurements

A detailed clinical history was obtained from each patient, including duration of illness, symptoms (fatiguability, bone pain, muscle weakness), comorbidities (diabetes mellitus, hypertension), and medication history. Baseline characteristics, including age, sex, blood pressure, and weight, were recorded. Laboratory investigations included complete blood count, random blood sugar, renal function tests, serum electrolytes (calcium, phosphorus), intact parathyroid hormone (iPTH), 25-hydroxyvitamin D, and serum albumin. Serum intact parathyroid hormone (iPTH) levels were measured using a second-generation chemiluminescent immunoassay at the institutional laboratory.

Assessment of bone mineral density

Bone mineral density was measured by DEXA at the lumbar spine, left femur, and right femur. T-scores were recorded at each site. Bone mineral density was categorized as normal, osteopenia, or osteoporosis using WHO criteria, and the overall category for each patient was assigned according to the lowest T-score observed among the measured sites [15]. Although KDIGO 2017 guidelines recommend the use of Z-scores in premenopausal women and men aged <50 years, T-scores were utilized in the present study to facilitate WHO-based categorization of osteopenia and osteoporosis and for comparison with prior studies. DEXA scans were performed using standard institutional protocols and routine machine calibration procedures as per manufacturer recommendations. However, detailed calibration logs and formal assessment of inter-observer variability were not systematically documented in this hospital-based study.

Statistical analysis

Statistical analysis was performed using IBM SPSS Statistics for Windows, version 28.0.11 (IBM Corp, Armonk, NY). Data were entered into Microsoft Excel (Microsoft Inc., Redmond, WA) and checked for accuracy before analysis.

Continuous variables were assessed for normality using the Shapiro-Wilk test. Since most continuous variables were not normally distributed, data are presented as median with interquartile range (IQR), and comparisons between CKD stage 3 and stage 4 were performed using the Mann-Whitney U test. Categorical variables were presented as frequencies and percentages and were compared using the chi-square test, and Fisher's exact test was used when expected cell counts were below five.

Associations between DEXA T-scores and biochemical markers of mineral and bone metabolism were assessed using Spearman's rank correlation coefficient. Correlation coefficients were interpreted according to the strength and direction of association between variables. Due to sample size considerations and the exploratory nature of the study, multivariable regression analysis was not performed.

All statistical tests were two-tailed, and a p-value <0.05 was considered statistically significant.

## Results

Baseline demographics

A total of 130 patients in CKD stages 3 and 4 were enrolled in the study. Of these, 72 (55.4%) were in Stage 3 and 58 (44.6%) were in Stage 4. The mean age of the study population was 47.9±14.3 years, with no significant difference between the stages.

**Figure 1:**
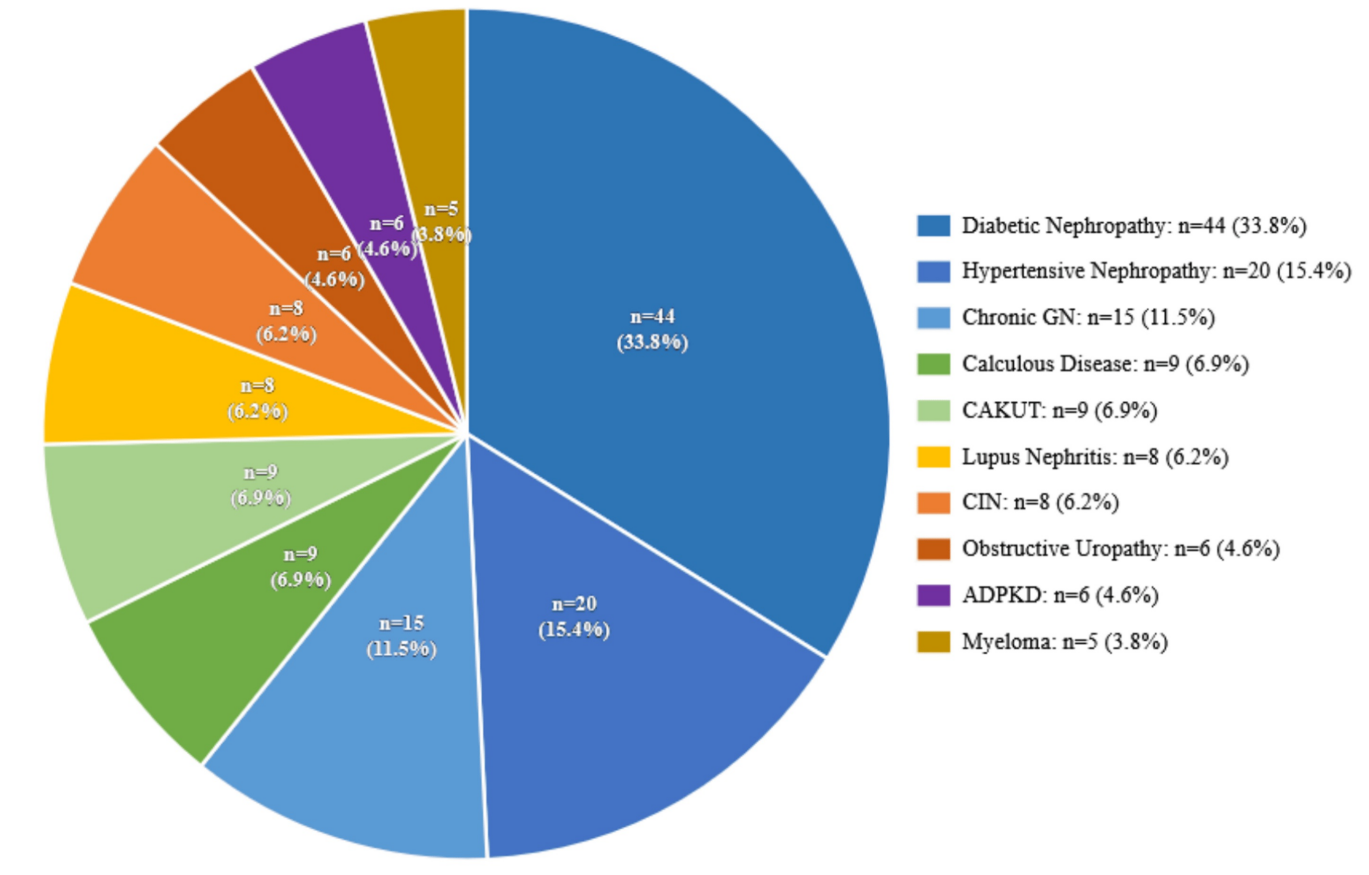
Distribution of CKD causes in the study population (n=130) ADPKD, autosomal dominant polycystic kidney disease; CAKUT, congenital anomalies of the kidney and urinary tract; CIN, chronic interstitial nephritis; CKD, chronic kidney disease; GN, glomerulonephritis Image credit: Figure created by the authors using MS Excel (Microsoft Corp., Redmond, WA, USA)

Diabetic nephropathy was the leading cause of CKD, accounting for 33.8% (n=44) of patients, followed by hypertensive nephropathy in 15.4% (n=20) and chronic glomerulonephritis in 11.5% (n=15). Other etiologies included calculous renal disease and congenital anomalies of the kidney and urinary tract (CAKUT) at 6.9% each, lupus nephritis and chronic interstitial nephritis at 6.2% each, and obstructive uropathy, autosomal dominant polycystic kidney disease (ADPKD), and multiple myeloma at 4.6%, 4.6%, and 3.8%, respectively. The mean eGFR was similar across etiological subgroups, ranging from 27.8 to 33.7 mL/min/1.73 m². The distribution of CKD causes is shown in Figure 1.

Biochemical profile

**Figure 2:**
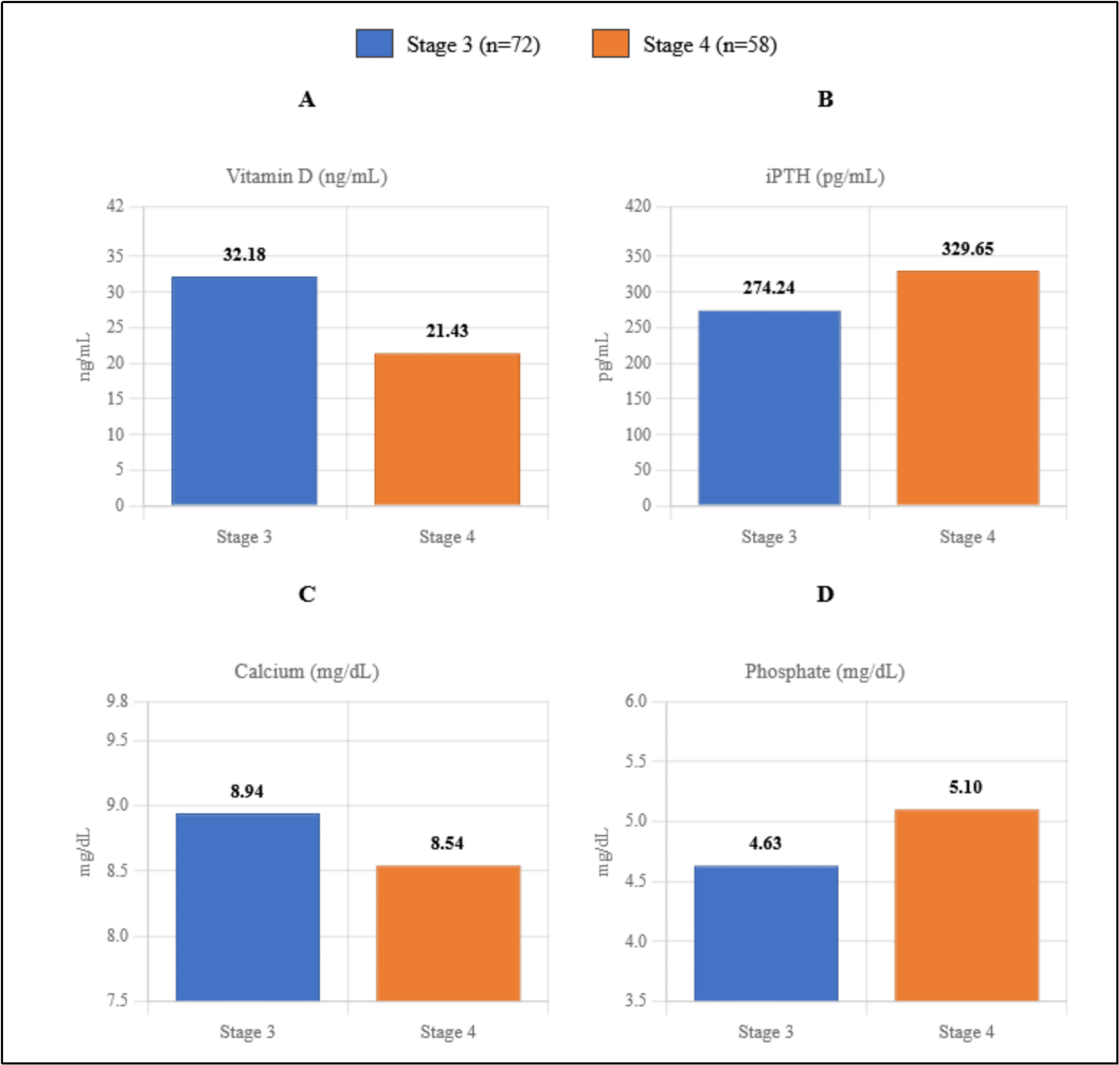
Biochemical parameters by CKD stage CKD, chronic kidney disease; iPTH, intact parathyroid hormone

The baseline biochemical parameters demonstrated significant differences between CKD stages. Stage 4 patients had higher serum creatinine (2.86±0.46 vs 1.90±0.29 mg/dL, p<0.0001) and lower eGFR (22.73±3.60 vs 38.95±6.48 mL/min/1.73 m², p<0.0001) than Stage 3 patients. Vitamin D levels were significantly lower in Stage 4 (21.43±6.82 vs 32.18±7.62 ng/mL, p<0.0001), while iPTH levels were higher (329.65±92.18 vs 274.24±84.14 pg/mL, p=0.0006). Serum calcium was lower in Stage 4 (8.54±0.77 vs 8.94±0.62 mg/dL, p=0.001), and serum phosphate was higher (5.10±0.81 vs 4.63±0.76 mg/dL, p=0.0011). The calcium-phosphate product was numerically higher in Stage 4 but did not reach statistical significance (43.56±7.81 vs 41.40±7.28, p=0.0699). These findings are detailed in Table 1. The biochemical parameters by CKD stage are depicted in Figure 2, while the T-score comparison by skeletal site and CKD stage is presented in Table 2.

**Table 1:** Comparison of biochemical parameters between CKD stages Values expressed as mean±SD; Mann-Whitney U test used for comparisons Ca × PO₄, calcium-phosphate product; CKD, chronic kidney disease ; eGFR, estimated glomerular filtration rate; iPTH, intact parathyroid hormone; SD, standard deviation.

Parameter	Overall (n=130)	Stage 3 (n=72)	Stage 4 (n=58)	U test, p-value
Creatinine (mg/dL)	2.33±0.61	1.90±0.29	2.86±0.46	(U=124.0) <0.0001
eGFR (mL/min/1.73 m²)	31.71±9.71	38.95±6.48	22.73±3.60	(U=4176.0) <0.0001
Vitamin D (ng/mL)	27.38±9.02	32.18±7.62	21.43±6.82	(U=3537.5) <0.0001
iPTH (pg/mL)	298.96±91.74	274.24±84.14	329.65±92.18	(U=1359.0) 0.0006
Calcium (mg/dL)	8.76±0.72	8.94±0.62	8.54±0.77	(U=2791.0) 0.001
Phosphate (mg/dL)	4.84±0.81	4.63±0.76	5.10±0.81	(U=1389.5) 0.0011
Ca × PO₄ (mg²/dL²)	42.36±7.57	41.40±7.28	43.56±7.81	(U=1700.5) 0.07

**Table 2:** T-score comparison by skeletal site and CKD stage Values expressed as mean±SD (range); Mann-Whitney U test used for comparisons CKD, chronic kidney disease; SD, standard deviation

Site	Overall (n=130)	Stage 3 (n=72)	Stage 4 (n=58)	p
Left Femur	-1.25 ± 1.35	-0.89 ± 1.37	-1.68 ± 1.20	(U=2849.5) 0.0004
Right Femur	-1.23 ± 1.33	-0.86 ± 1.28	-1.69 ± 1.26	(U=2988.5) <0.0001
Lumbar Spine	-1.57 ± 1.48	-1.22 ± 1.45	-2.01 ± 1.42	(U=2856.5) 0.0003

BMD categories

**Figure 3:**
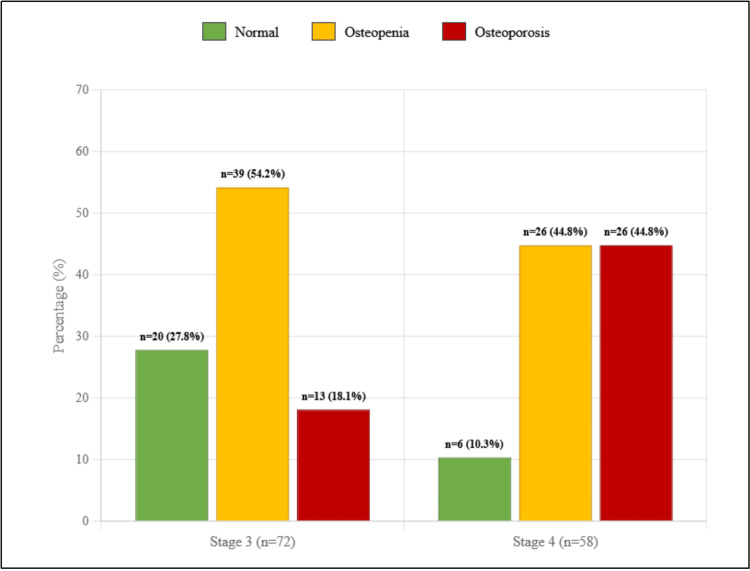
BMD category distribution by CKD stage BMD, bone mineral density; CKD, chronic kidney disease

Based on the worst T-score across all the measured sites, only 20.0% (n=26) of patients had normal bone density. Osteopenia was present in 50% (n=65), while osteoporosis was identified in 30% (n=39). The distribution differed significantly across CKD stages: normal BMD was observed in 27.8% of Stage 3 versus only 10.3% of Stage 4 patients. Osteoporosis prevalence was markedly higher in Stage 4 (44.8%) compared to Stage 3 (18.1%) (Chi-square p=0.0014). These findings are summarized in Table 3 and Figure 3.

**Table 3:** BMD category distribution by CKD stage Chi-square p=0.0014 for distribution between stages BMD, bone mineral density; CKD, chronic kidney disease; T, T-score

BMD Category	Overall (n=130)	Stage 3 (n=72)	Stage 4 (n=58)
Normal (T≥-1.0)	26 (20.0%)	20 (27.8%)	6 (10.3%)
Osteopenia (-2.5	65 (50.0%)	39 (54.2%)	26 (44.8%)
Osteoporosis (T≤-2.5)	39 (30.0%)	13 (18.1%)	26 (44.8%)

Correlation analysis

Spearman correlation analysis revealed significant associations between T-scores and several biochemical parameters. eGFR demonstrated a moderate positive correlation with T-scores at all sites (ρ=0.32-0.40, p≤0.0002), suggesting an association between renal function and bone density. Serum calcium showed the strongest, though moderate, positive biochemical correlation with T-scores (ρ=0.30-0.41, p≤0.0004). Vitamin D exhibited weak but significant positive correlations (ρ=0.20-0.26, p<0.03). Serum creatinine and age showed negative correlations with T-scores. iPTH showed weak negative associations that did not reach statistical significance (ρ=-0.12 to -0.15, p>0.10). The calcium-phosphate product showed no meaningful correlation with T-scores. Table 4 summarizes the correlation data.

**Table 4:** Correlation of T-scores with biochemical parameters ρ: Spearman correlation coefficient; *p<0.05; **p<0.01. eGFR; estimated glomerular filtration rate; iPTH, intact parathyroid hormone; Vit D, 25-hydroxyvitamin D.

Site	eGFR	Calcium	Phosphate	Vit D	iPTH	Age
Left Femur (ρ)	0.32**	0.30**	-0.14	0.20*	-0.15	-0.30**
Right Femur (ρ)	0.40**	0.37**	-0.23*	0.26*	-0.14	-0.28**
Lumbar Spine (ρ)	0.34**	0.41**	-0.20*	0.24*	-0.12	-0.22*

## Discussion

In this single-centre cohort of 130 CKD patients, our findings demonstrated a high prevalence of CKD-MBD and reduced bone mineral density in this pre-dialysis population, with progressive worsening of both biochemical and skeletal parameters as kidney function declines.

The demographic profile of our cohort showed a mean age of 47.9 years with male predominance (59.2%), which is consistent with patterns observed in other Indian studies. Vikrant and Parashar (2016) reported similar findings in their study of 462 CKD patients across stages 3-5D, with a mean age of 56.8 years and 56.9% of the participants being men [12]. Diabetic nephropathy emerged as the leading etiology (33.8%) in our study, followed by hypertensive nephropathy (15.4%), reflecting the epidemiological transition in CKD causation in India. This pattern aligns with global trends and was similarly observed by Vikrant and Parashar (2016), who found diabetic nephropathy in 27.7% of their cohort [12].

Our biochemical analysis revealed a clear gradient of mineral metabolism derangement between CKD stages 3 and 4. Stage 4 patients demonstrated significantly lower vitamin D levels (21.43 vs 32.18 ng/mL), higher iPTH (329.65 vs 274.24 pg/mL), lower serum calcium, and higher phosphate compared to Stage 3. These findings are consistent with the progressive nature of CKD-MBD as documented in international literature. Ghosh et al. (2012) reported that the prevalence of hyperphosphataemia, hypocalcaemia, and secondary hyperparathyroidism increased significantly between CKD stages 4 and 5 [13]. Vikrant and Parashar (2016) similarly observed that mean iPTH increased progressively from 159 pg/mL in stage 3 to 320 pg/mL in stage 4 and 469 pg/mL in stage 5 [12].

The DEXA findings in our study are particularly noteworthy. We observed significantly lower T-scores in Stage 4 patients across all measured sites (left femur, right femur, and lumbar spine), with all comparisons reaching statistical significance (p≤0.0004). The mean lumbar spine T-score was -1.22 in Stage 3 and -2.01 in Stage 4. These findings are comparable to those reported by Nickolas et al. (2011), who found that CKD patients with fractures had significantly lower femoral neck T-scores (-2.2 vs -1.4) compared to those without fractures [14]. Tariq and Sulaiman (2020), in their systematic review, reported T-scores at the femur ranging from -1.0 to -1.8 and lumbar spine T-scores between -1.1 and -1.7 across various CKD cohorts [15]. However, although DEXA provides a useful assessment of bone mineral density, it cannot distinguish between high-turnover and low-turnover renal bone disease, which limits its ability to fully characterize CKD-MBD skeletal pathology.

The prevalence of osteoporosis in our cohort was 30.0% overall, with a striking difference between stages: 18.1% in Stage 3 versus 44.8% in Stage 4 (p=0.0014). This represents a more than twofold increase in osteoporosis prevalence with advancing CKD stage. Tariq and Sulaiman (2020) reported osteoporosis prevalence ranging from 2.24% to 31.3% across eight studies in CKD stages 3-5 [15]. Fidan et al. (2016) found osteoporosis in 31.3% of non-dialysis CKD patients, while Aggarwal et al. (2013) reported 21.3% in pre-dialysis CKD stages 3-5 [16,17]. The higher prevalence in our Stage 4 cohort underscores the accelerated bone loss occurring in this population. The observed reduction in BMD may suggest potential skeletal vulnerability in CKD patients; however, fracture risk assessment using FRAX or CKD-specific tools was not performed in the present study.

The correlation analysis in our study revealed that eGFR was positively correlated with T-scores (ρ=0.32-0.40), confirming that better renal function is associated with higher bone density. Serum calcium showed the strongest biochemical correlation with T-scores (ρ=0.30-0.41). These findings are supported by Uhlinova et al. (2022), who reported a positive correlation between eGFR and BMD at multiple sites (p=0.003-0.04), as well as an inverse correlation between iPTH and BMD [18]. Interestingly, iPTH showed only weak, non-significant negative correlations with T-scores in our study (ρ=-0.12 to -0.15), which aligns with findings from Baszko-Blaszyk et al. (2001), who also found weak or non-significant associations between iPTH and BMD [19]. Although iPTH levels were significantly higher in CKD Stage 4, no significant correlation with T-scores was observed. This may reflect the multifactorial determinants of bone mineral density in CKD-MBD and the limited ability of single-point iPTH measurements to fully capture bone turnover status. Additionally, skeletal abnormalities in CKD are influenced by multiple factors, including phosphate retention, vitamin D deficiency, inflammation, and alterations in bone remodeling.

The progressive deterioration in bone density observed in our cohort is likely multifactorial. The pathophysiology of CKD-MBD involves complex interactions between phosphate retention, secondary hyperparathyroidism, vitamin D deficiency, and FGF-23/Klotho axis dysregulation [20,21]. As kidney function declines, reduced phosphate excretion and impaired vitamin D activation lead to hypocalcaemia and compensatory PTH elevation, promoting bone resorption. Our findings of lower vitamin D and higher iPTH in Stage 4 supported this mechanism.

The clinical implications of our findings are significant. The high prevalence of osteopenia (50%) and osteoporosis (30%) in our cohort suggests that DEXA-based BMD assessment may be useful in selected pre-dialysis CKD patients, particularly those at higher risk of CKD-MBD. Nickolas et al. (2013) demonstrated annual BMD declines of -1.3% at the total hip and -2.4% at the ultradistal radius in CKD stages 2-5D, highlighting the progressive nature of bone loss [22]. However, given the cross-sectional nature of our study and the absence of fracture outcomes or cost-effectiveness analyses, our findings should be interpreted with cautioun. Larger prospective studies are needed before recommending routine BMD screening for all CKD stage 3-4 patients.

Strengths of the study

Our study provided valuable data on CKD-MBD in a pre-dialysis Northeast Indian population, a group that has been underrepresented in the literature. The integration of biochemical profiling with DEXA-based skeletal assessment offered a comprehensive approach to evaluating bone health in CKD patients.

Limitations of the study

Our study has several limitations. As this was a hospital-based cross-sectional study, detailed screening logs, including the number of eligible patients screened and those declining participation, were not systematically maintained, limiting the assessment of response or enrollment rates. The single-centre design and moderate sample size may limit the generalizability of findings. The cross-sectional nature of the study precluded assessment of temporal relationships, longitudinal changes in bone mineral density, or fracture outcomes. Additionally, multivariable adjustment for potential confounders such as age, sex, diabetes status, and BMI was not performed, limiting the assessment of the independent contribution of CKD stage to reduced BMD. DEXA measurements were restricted to three skeletal sites, and vertebral fractures were not systematically assessed. Although DEXA scans were performed using standard institutional protocols and routine machine calibration procedures, formal assessment of inter-operator and inter-machine variability was not systematically documented, which may have introduced measurement variability. Furthermore, we did not measure fibroblast growth factor 23 (FGF-23), bone turnover markers, or assess vascular calcification, which may have provided additional insights into the complex pathophysiology of CKD-MBD. Future multicentre prospective studies with longer follow-up are needed to better characterize bone disease progression and fracture risk in CKD patients.

## Conclusions

Mineral and bone disorder is a clinically important complication in patients with CKD stages 3 and 4. Our study demonstrates that advancing CKD stage is associated with worsening biochemical abnormalities and declining bone mineral density, with a higher prevalence of osteopenia and osteoporosis observed in stage 4 compared to stage 3 CKD. DEXA-derived bone mineral density assessment may provide an objective measure of skeletal involvement and could complement biochemical evaluation in CKD-MBD. These findings highlight the association between CKD progression and bone health deterioration, warranting further longitudinal studies to determine the predictive and therapeutic implications of bone mineral density assessment in CKD patients.
